# Railway Injuries: In Their Medico-Legal and Clinical Aspects

**Published:** 1892-06

**Authors:** 


### Reviews of Books.

*Railway Injuries: in their Medico-Legal and Clinical Aspects.* By HERBERT W. PAGE. Pp. 148. London: CHARLES GRIFFIN AND COMPANY. 1891.

A new book by the author of *Injuries of the Spine and Spinal Cord and Nervous Shock*, which threw so much fresh light on these maladies that were till then so ill-understood, will be taken up with expectations in which its careful reader will not be disappointed. Mr. Page has the satisfaction of finding that the views which he then expressed as to the nature and pathogeny of these affections, and which were in strong divergence from those most widely prevalent at that time (1883), have met with general acceptance not only in this country, but by foreign writers also. His subsequent experience has served to confirm his former opinions. The change of view that has taken place with regard to the real nature of the nervous symptoms produced by a severe accident, and the paramount importance that is now attributed to the influence of "nervous shock," is not the least remarkable of the recent advances in neurology. Whereas the older writers looked for the cause of these symptoms in morbid changes of greater or less intensity, produced in the central nervous system by concussion or some similar agency, according to the modern view they are due, not to organic changes, but to functional disturbances, in which the psychical element plays an important part, acting by means of the condition of "nervous shock" that is evoked by the general consternation that follows upon the appalling suddenness and attendant horrors of a bad railway accident. Further, the researches into the nature of the nervous malady excited by traumatisms have shown that in many cases it is identical with hysteria arising from other causes. As a matter of fact, Mr. Page finds actual organic lesions of the spinal cord to be rarely produced, although painful bruises and sprains of the spinal ligaments and muscles may easily lead, unless due care be taken, to mistakes in diagnosis. The book contains a wealth of apt clinical illustrations drawn from the author's large experience in these cases. The subject of the influence of litigation for compensation on the progress of an illness brought about by a railway accident, and the manner in which it may retard recovery in cases in which there is no reason to doubt the *bona fides* of the patient, and the other important one of the detection of malingerers, are very ably considered. If all practitioners were possessed of the knowledge that is accessible to them in this book, we should not so often find those unfortunate differences of opinion amongst medical witnesses in suits for damages against railway companies, which stultify the profession in the eyes of the public. We have said enough to indicate the interesting nature of the subject which Mr. Page treats in a singularly lucid and able manner, and can only add that his book is one to be read and digested.

